# POLQ immunostaining behaves as a prognostic factor for pancreatic carcinoma

**DOI:** 10.3389/fonc.2024.1433179

**Published:** 2024-10-07

**Authors:** Laura del Puerto-Nevado, María J. Fernández-Aceñero, Arancha Cebrián, Yuliia Fatych, Luis I. Díez-Valladares, Elia Pérez-Aguirre, Sofía de la Serna, Alejandra García-Botella, Javier Martínez-Useros, Jesús García-Foncillas, Pedro A. Mateos-Gómez

**Affiliations:** ^1^ Instituto de Investigación Sanitaria Fundación Jiménez Díaz (IIS-FJD), Universidad Autónoma de Madrid (UAM), Madrid, Spain; ^2^ Pathology Department, Hospital Clínico San Carlos, Madrid, Spain; ^3^ Biochemistry and Molecular Biology Unit, Systems Biology Department, School of Medicine and Health Sciences, University of Alcalá, Madrid, Spain; ^4^ Hepatobiliary Unit, Surgery Department, Hospital Clínico San Carlos, Madrid, Spain

**Keywords:** POLQ, pancreatic carcinoma, immunostaining, overall survival, prognostic factor

## Abstract

**Background:**

DNA polymerase theta (POLQ) is a translesion synthesis polymerase essential for the repair of double strand breaks by the error-prone TMEJ (Theta Mediated End Joining) pathway. Although POLQ participates in maintaining genome stability, several studies have shown that its overexpression correlates with cancer progression and poor prognosis. Due to the fact that its role as a biomarker in pancreatic cancer remains unexplored, we aimed to study the usefulness of POLQ H-score as a prognostic factor in a pancreatic cancer patient cohort.

**Methods:**

We evaluated *POLQ* gene expression using a web-based tool to deliver gene expression profiling and interactive analyses based on TCGA and GTEx (GEPIA) and we examined the POLQ immunostaining in 152 biliopancreatic cancer surgical specimens using tissue microarrays. Association with survival was evaluated by Kaplan Meier curves and uni-multivariate Cox regression.

**Results:**

GEPIA analysis showed statistical differences according to *POLQ* mRNA levels in Disease Free Survival (DFS) (log rank 0.023, HR 2.8, *p=*0.029) and Overall Survival (OS) (log rank 0.011, HR 3.1, *p=*0.016). For immunohistochemistry (IHC) evaluation, POLQ H-score was calculated, and showed statistical differences for OS in Kaplan Meier curves (log rank 0.001) and uni-multivariate analysis (HR 2.27; 95% CI 1.24-4.15, *p*=0.008).

**Conclusions:**

Our results indicate that POLQ is an independent prognostic factor in pancreatic cancer when analyzed by immunostaining, which is in agreement with the results shown by the *POLQ* gene expression analysis (GEPIA).

## 1 Introduction

Pancreatic cancer is one of the most aggressive human tumors because of its rapid growth and its ability to form metastases. Due to these characteristics, diagnosis is usually only possible at advanced stages, leading to a high mortality rate. Despite its relatively low incidence, pancreatic cancer ranks seventh in terms of cancer-related death worldwide (1). Only 20% of patients with pancreatic cancer present surgically resectable tumors at diagnosis, and despite successful surgical resection, 5-year survival remains at 27% (2). Hence, it is important to evaluate new strategies to improve the management and outcome of these patients, concentrating the efforts on the search for valuable biomarkers that could also have therapeutic impact for its clinical application.

DNA replication stress and genomic instability are one of the hallmarks of cancer, helping tumor cells to escape from cell death under uncontrolled proliferation, what makes targeting the DNA damage response (DDR) an effective antitumoral strategy (3, 4). Therefore, the cellular pathways for DNA repair are one of the fields of intense research, of course also related to pancreatic cancer.

Among the DNA repair mechanisms, there is a double strand breaks (DSBs) repair pathway in which the DNA polymerase theta (POLQ) has an essential role, the Theta Mediated End Joining (TMEJ) (5, 6). This is a protein encoded by the *POLQ* gene and this pathway is crucial for some cancers with deficiencies in homologous recombination (HR), given their reported dependence on TMEJ for survival and tumor growth (5, 7). In addition, POLQ depletion sensitizes cells to several agents that induce DSBs such as camptothecin, bleomycin, etoposide and irradiation (8).

POLQ is a unique DNA polymerase with an N-terminal helicase-like domain, a central domain with no similarities to other enzymes, and a C-terminal A-family DNA polymerase domain (9, 10). Recently it was confirmed that POLQ can dimerize to facilitate the approximation of DNA ends in order to form synapsed intermediates using microhomologies in the sequence, which is the previous step for DNA synthesis (11). In normal cells POLQ is absent or its activity is low, and *polq* knock-out has a minor impact on development. In addition to its previously reported requirement for survival in BRCA1/2 mutated cells, POLQ would protect cancer cells with functional canonical DSBs repair mechanism from the accumulation of DNA replication associated DSBs (12).

Several studies have shown the association of POLQ expression with outcome in other tumor entities such breast cancer (13, 14), ovarian (7) colorectal cancer (15) or lung cancer (16), and also its relation with pathogenesis in lung adenocarcinoma (17, 18), hepatocellular carcinoma (19) and esophageal squamous cell carcinoma (20, 21). Very recently, the involvement of TMEJ has been implicated in the development and metastasis of KRAS mutated pancreatic cancer (22) and its expression has been associated with a better response to immunotherapy in a subset of bladder cancer patients (23). Additionally, POLQ inhibition in this type of cancer with BRCA2 deficiency is synthetically lethal as expected, but also stimulates the immune response (24).

Since GEPIA (Gene Expression Profiling Interactive Analysis) (25) data showed differences in expression and patient survival, we decided to study whether this DNA polymerase could serve as biomarker in pancreatic cancer. Despite evidence reported in other tumors, there is little data regarding either the impact of POLQ in pancreatic cancer, or the usefulness of immunohistochemistry staining of POLQ as a valuable technique to associate its expression level with patient outcome.

In this report we evaluate the usefulness of POLQ expression analysis for outcome prediction in pancreatic cancer. Additionally, we tested the potential of POLQ immunostaining as a valid technique for this analysis in formalin-fixed, paraffin-embedded (FFPE) tissue sections. Our results indicate that the POLQ H-score obtained from immunohistochemistry is a valuable prognostic biomarker for pancreatic cancer outcomes, which may also contribute to the future design of more personalized therapies that improve patient prognoses.

## 2 Methods

### 2.1 GEPIA analysis

The GEPIA (Gene Expression Profiling Interactive Analysis) website (http://gepia.cancer-pku.cn/) offers an online tool for analyzing the RNA sequencing expression data of 9,736 tumors from the TCGA and GTEx projects, using a standard processing pipeline. GEPIA allows many interactive and customizable functions including patient survival analysis (25). In our study, GEPIA was used to obtain data about the potential impact of *POLQ* gene expression in terms DFS and OS in pancreatic adenocarcinoma patients. For both analyses we used a cut off point for *POLQ* expression of 10%.

### 2.2 Patients

A total of 182 biliopancreatic cancer patients who underwent surgery from 2006 to 2012 at the Surgery Department of University Hospital Clínico San Carlos were included in the database. Patients were followed until March 2019 and their clinicopathological characteristics regarding tumor location, stage, histologic type, tumor grade, tumor size, vessel invasion, perineural infiltration and margin status were recorded. The outcome measurements were progression-free survival, defined as the time in months between surgery with a curative intent and disease recurrence shown with imaging methods; and overall survival, defined as the time in months elapsed between surgery with a curative intent and death due to disease.

### 2.3 Tissue sampling

FFPE samples from 182 biliopancreatic cancer surgical specimens were used for tissue microarray (TMA) construction. Representative tumor regions from samples were identified by a pathologist (MJFA) on hematoxylin- and eosin-stained tissue sections. After pathologist review, TMAs were assembled from triplicate 0.6-mm cores of FFPE tumor samples using the TMA workstation MTA-1 (Beecher Instruments).

### 2.4 Immunohistochemistry staining

For the immunohistochemical techniques the antigen retrieval was performed in a PT-Link (Dako, Glostrup, Denmark) for 20 min at 95 °C in citrate buffer (Dako). Endogenous peroxidase was blocked by adding 0.03% hydrogen peroxide for 5 min. Slides were then washed for 5 min with Tris-buffered saline solution containing Tween 20 at pH 7.6 and incubated with the primary antibody (1:100 POLQ, Thermofisher PA5-115130) for 1.5 hours at room temperature, followed by 30 min incubation with the appropriate anti-Ig horseradish peroxidase-conjugated polymer (EnVision, Dako) to detect antigen–antibody. Sections were then visualized with 3,3′-diaminobenzidine as a chromogen and counterstained with haematoxylin. Only 152 patient samples reached enough quality for immunohistochemical evaluation.

We scanned the TMA stained slide at low power and selected the case showing the highest intensity of staining, which was graded as 3. Then we chose two patterns of staining of moderate (graded as 2) and weak intensity (graded as 1) in the cases, observing a negative staining pattern as well. After marking these cases as reference, we compared all the other cores with the references for grading. Once the grade of each case was assigned, the Histoscore (H-score) of POLQ nuclear staining was determined as the percentage of positive cells in every case multiplied by the intensity of the grade assigned (0=negative; 1=weak; 2=moderate; 3=strong); hence the H-score values could range between 0 and 300.

The cases were blindly graded. Pathologist was unaware of the outcome of the patients.

### 2.5 Statistical analyses

Clinicopathological patients’ characteristics were reported as frequency (and percentage). The relationship between POLQ H-score and survival (DFS and OS) was estimated using the Kaplan Meier method and significant survival differences between groups were determined by the log rank test.

The relationship between each variable and survival (DFS and OS) was investigated by resorting to a Cox proportional hazards regression model in both univariate and multivariate fashion. Those variables that had potential prognostic significance in univariate analysis (significant variables or variables trending to significance) were subjected to multivariate analysis.

The H-score cutoff point used for this analysis was established in first quartile (Low POLQ H-score ≤ 10 vs High POLQ H-score>10). A *p*-value < 0.05 was considered statistically significant. All statistical analysis was performed using SPSS software version 20.0 (SPSS Inc., Chicago, IL).

## 3 Results

### 3.1 Higher *POLQ* gene expression associates with lower patient survival with pancreatic adenocarcinoma

**Figure 1:**
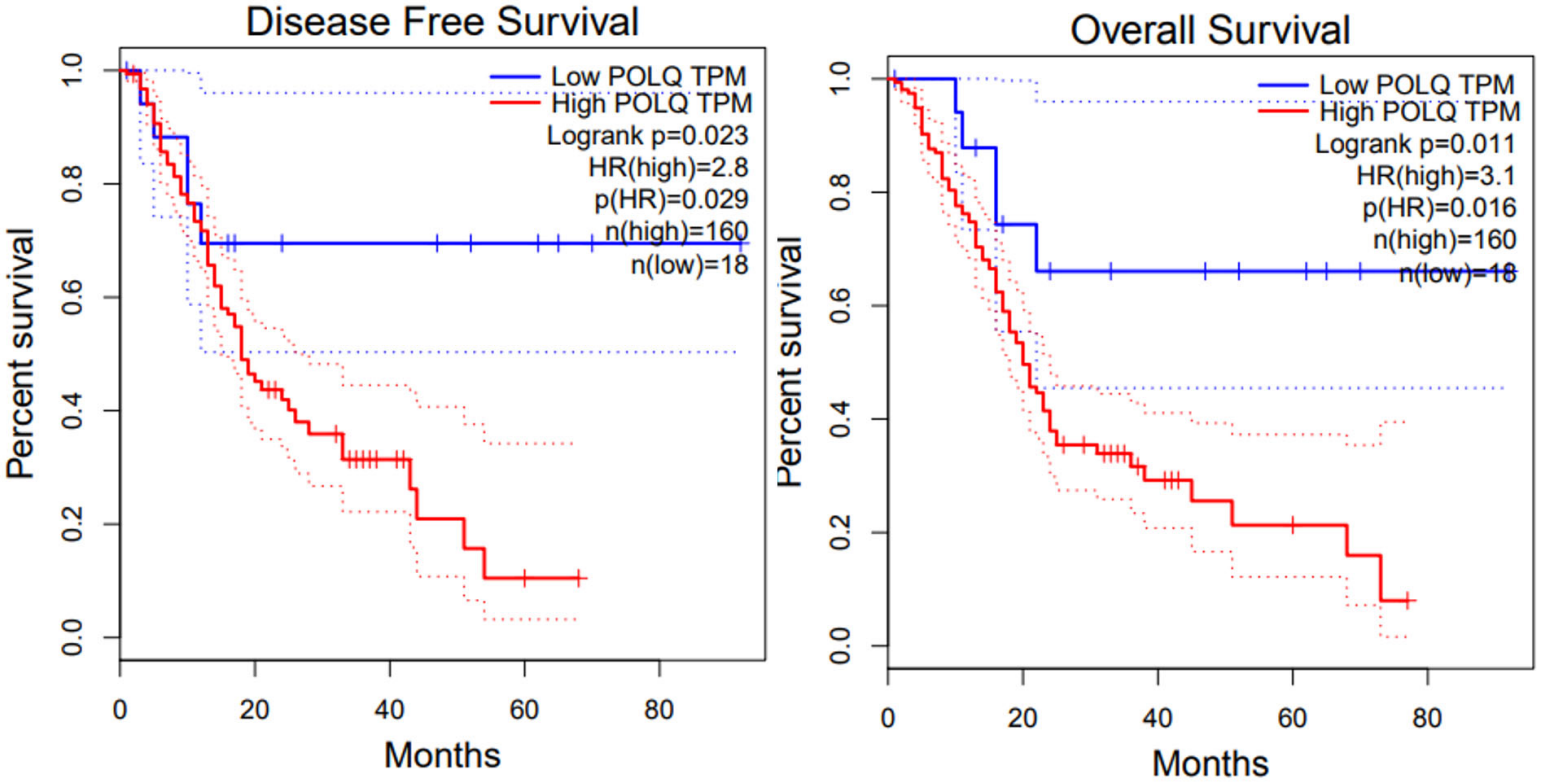
*POLQ* mRNA expression is upregulated in pancreatic adenocarcinoma and associated with survival. The expression of *POLQ* in pancreatic adenocarcinoma tissues was collected from TCGA database. Kaplan Meier survival analysis of patients with low and high *POLQ* mRNA expression using the Mantel–Cox [log rank] test, n = 178; p value is indicated for comparison of patients with higher or lower *POLQ* expression. In the left is shown the analysis for disease free survival and in the right is shown the analysis for overall free survival.

To assess the prognostic potential of *POLQ* gene expression in patients with pancreatic adenocarcinoma, we conducted a survival analysis using Gene Expression Profiling Interactive Analysis (GEPIA) (25). We studied the prognostic impact of *POLQ* gene expression in terms of PFS and OS in these samples (N=178) using a cutoff point of 10%. Results obtained with Kaplan Meier method were the following: log-rank=0.023, Hazard Ratio=2.8 and *p*-value 0.029 for PFS and log-rank=0.011, Hazard Ratio=3.1 and *p*-value 0.016 for OS, suggesting a predictive and prognostic role of *POLQ* gene expression in this group of patients (**Figure 1**).

### 3.2 Data analysis of a biliopancreatic cancer cohort

We conducted a retrospective study with cohort of 152 patients with resectable biliopancreatic cancer, comprising only cancer stages I and II. In **Table 1** we summarized the baseline patient’s characteristics. Most patients included were over 60 years old (79.6%). The distribution of sex across the cohort was similar (47.4% males vs 52.6% females). The predominant tumor location was pancreas and bile duct with few cases of ampullary carcinomas (13.2%); most neoplasms were ductal adenocarcinomas (73%). Regarding sample characteristics, most of the tumors were categorized as high grade (59.9%), and the size of 48.7% of them was over 20 mm. Around half of the tumors (48.7%) presented desmoplastic stroma and 29.6% showed positive disease resection margins in the microscopic analysis, mainly the superior mesenteric vein margin (R1 disease). Regarding tumor aggressive features, 39.5% of patients presented vascular invasion and 65.8% perineural infiltration. Due to the lack of standardization of adjuvant therapy after surgery in this kind of tumor, only 34.9% of patients received adjuvant treatment.

**Table 1:** Baseline characteristics of included patients.

VARIABLE	PATIENTS (N=152)
Age	
≤60	*31 (20.4%)*
>60	*121 (79.6%)*
Sex	
Male	*72 (47.4%)*
Female	*80 (52.6%)*
Localization	
Pancreas & bile duct	*130 (85.5%)*
Ampulla	*20 (13.2%)*
*N/A*	*2 (1.3%)*
Histology	
Intestinal	*21 (13.8%)*
Ductal	*111 (73%)*
Others	*19 (12.5%)*
*N/A*	*1 (0.7%)*
Stage	
I	*50 (32.9%)*
II	*91 (59.9%)*
*N/A*	*11 (7.2%)*
Tumor grade	
Low grade	*58 (38.2%)*
High grade	*91 (59.9%)*
*N/A*	*3 (2%)*
Tumor size (mm)	
≤20	*49 (32.2%)*
>20	*74(48.7%)*
*N/A*	*29 (19.1%)*
Desmoplasia	
No	*69 (45.4%)*
Yes	*74 (48.7%)*
*N/A*	*9 (5.9%)*
Resection margin	
Negative	*95 (62.5%)*
Positive	*45 (29.6%)*
*N/A*	*12 (7.9%)*
Vascular invasion	
No	*84(55.3%)*
Yes	*60 (39.5%)*
*N/A*	*8 (5.3%)*
Perineural invasion	
No	*45 (29.6%)*
Yes	*100 (65.8%)*
*N/A*	*7 (4.6%)*
Adyuvance	
No	*77 (50.7%)*
Yes	*53 (34.9%)*
*N/A*	*22 (14.5%)*

N/A, Not available; mm, millimeters.

### 3.3 POLQ immunostaining of pancreatic tumor samples showed different degrees of protein expression

POLQ protein expression was assessed by immunohistochemistry. The final value to calculate H-score was stablished in terms of percentage of positive cells and staining intensity for each case, evaluating samples in triplicate and representing the value as the mean of the three results.

**Figure 2:**
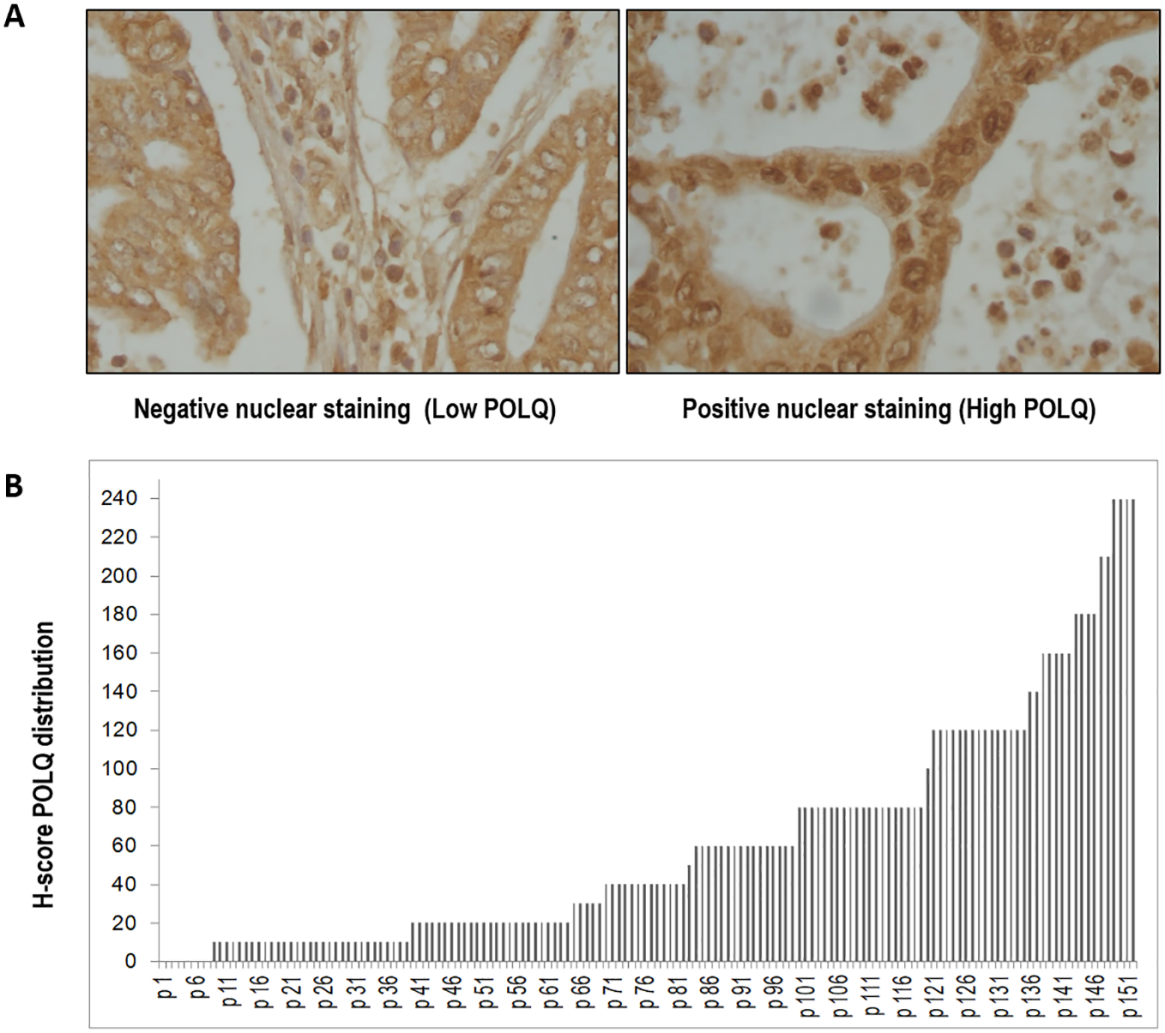
The expression of POLQ in pancreatic adenocarcinoma tissues was detected by IHC. **(A)** representative images of pancreatic tissues after POLQ immunostaining (x400 magnification). **(B)** distribution of POLQ H-scores of pancreatic adenocarcinoma tissues analyzed. H-score of POLQ nuclear staining was determined as the percentage of positive cells multiplied by the intensity of the case (0=negative; 1=weak; 2=moderate; 3=strong); hence the H-score values range between 0 and 300.

We also examined the expression localization pattern of POLQ, that was detected in the nucleus and the cytoplasm as shown in **Figure 2A**. However, considering its role in TMEJ, only nuclear staining was recorded for later H-score calculation. After POLQ expression analysis H-scores were calculated and plotted in a histogram. The H-score values obtained from the cohort revealed a median interquartile range (IQR) value of 40 (10, 80) (**Figure 2B**).

### 3.4 POLQ nuclear staining predicts clinical outcome in patients with pancreatic adenocarcinoma

**Figure 3:**
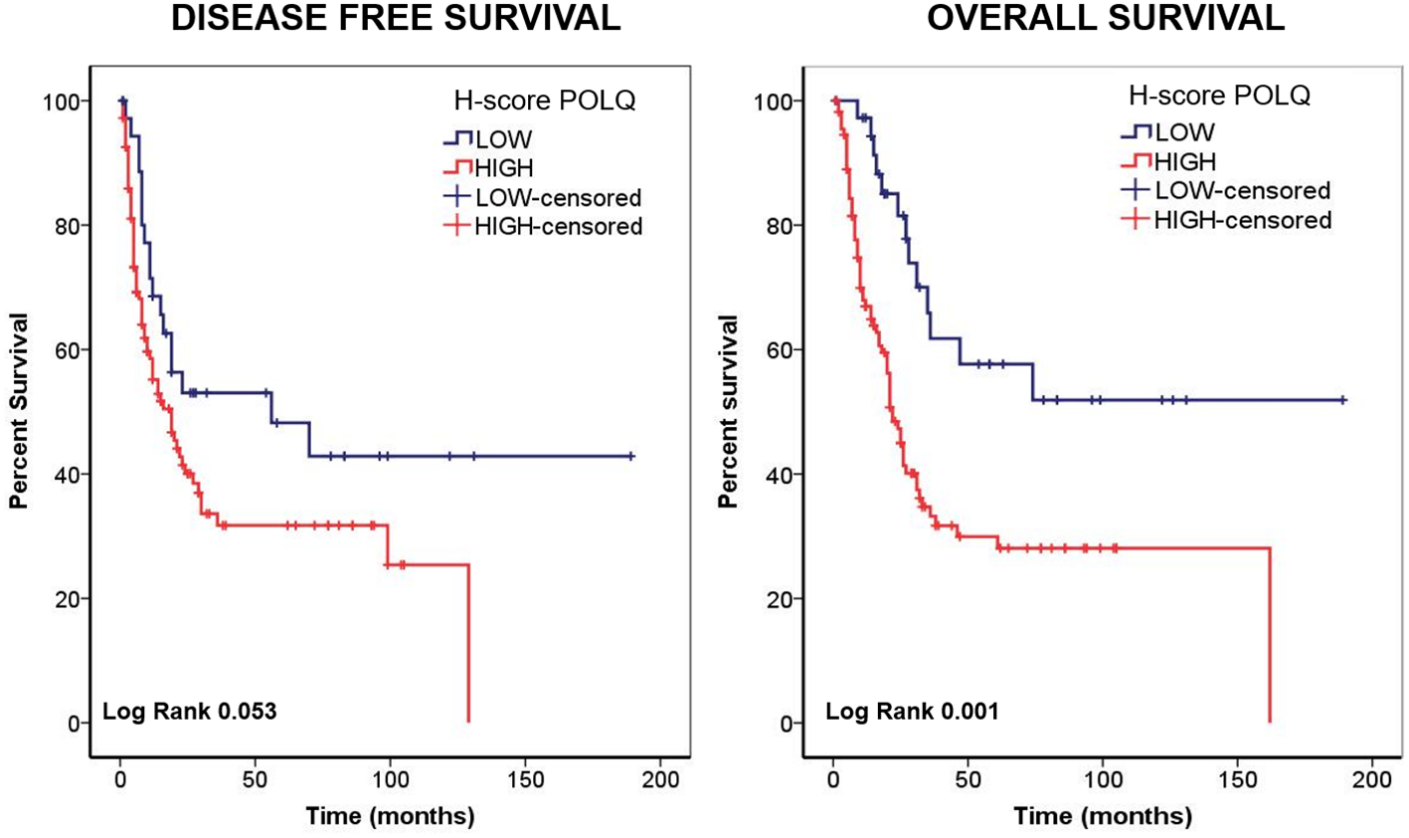
The expression of POLQ in pancreatic adenocarcinoma tissues detected by IHC predicts patient survival. The relationship between POLQ expression in pancreatic adenocarcinoma tissues detected by IHC and patient survival analyzed by Kaplan Meier. In the left is shown the analysis for disease free survival and in the right is shown the analysis for overall survival.

In terms of overall survival, log rank test showed statistical differences for the staining of this biomarker (log rank. 0.001). The median survival time for the patients with low POLQ was 28 months (IQR, 16 – 74) whereas those cases with high POLQ, showed a worse outcome, with a significantly lower median survival time of 17 months for OS (IQR, 7.5 – 31.5) (**Figure 3**). These results were in agreement with those obtained with GEPIA and *POLQ* gene expression.

Based on the cutoff point established for POLQ H-score nuclear staining, Kaplan Meier analysis for POLQ as marker was performed in terms of disease-free survival and overall survival, and the correlation between the expression levels of POLQ and prognosis status in patients with pancreatic adenocarcinoma was determined. Regarding the first parameter evaluated, DFS, log rank test derived from Kaplan Meier curve showed a nonsignificant, but strong trend to significance (log rank. 0.053); the median survival time for the patients with low POLQ was 19 months (interquartile range, IQR, 9 – 58) whereas those cases with high POLQ, presented a median survival time of 10 for OS (IQR, 5 – 25).

Considering the results obtained in Kaplan Meier curves, Cox regression was also performed for DFS and OS; multivariate analysis did not show statistical significance for DFS as expected (Data not shown).

However, results for OS were more robust. In univariate analysis, stage and perineural infiltration were significant (HR, 1.66; 95% CI 1.02-2.71, p = 0.04 and HR, 2.21 95% CI 1.29-3.78, p = 0.004, respectively). Sex, histology and tumor grade showed a trend to significance (HR, 1.56; 95% CI 0.99-2.45, p=0.05; HR, 2.08; 95% CI 0.99-4.34, p=0.05 and HR,1.56; 95% CI 0.98-2.49, p=0.06). The molecular marker, POLQ, also showed statistical significance in univariate analysis, (HR, 2.62; 95% CI 1.44-4.76, p=0.002).

As shown in **Table 2**, in multivariate analysis, performed with univariate significant variables, only the result for POLQ H-score remained statistically significant (HR, 2.27; 95% CI 1.24-4.15, p=0.008), suggesting that POLQ could be an independent predictive factor of outcome for patients with biliopancreatic cancer, in agreement with the results of the *POLQ* gene expression analysis (GEPIA).

**Table 2:** Analysis of the relation between POLQ H-score and tumor characteristics in patients with pancreatic adenocarcinoma by uni and multivariate analysis for overall survival.

Variables	OS Univariate		OS Multivariate	
	HR (IC)	*P* value	HR (IC)	*P* value
Age				
≤60	1	*0.23*		
>60	1.39 (0.80-2.41)			
Gender				
Male	1	*0.05*		
Female	1.56 (0.99-2.45)			
Localization				
Pancreas & bile duct	1	*0.09*		
Ampulla	1.87 (0.90-3.90)			
Histology				
Intestinal	1	*0.05*		
Ductal	2.08 (0.99-4.34)			
Stage				
I	1	***0.04***	1	*0.07*
II	1.66 (1.02-2.71)		1.62 (0.95-2.74)	
Tumor grade				
Low grade	1	*0.06*		
High grade	1.56 (0.98-2.49)			
Tumor size (mm)				
≤20	1	*0.72*		
>20	1.1 (0.66-1.82)			
Desmoplasia				
No	1	*0.95*		
Yes	1.01 (0.65-1.59)			
Resection margin				
Negative	1	*0.12*		
Positive	1.48 (0.90-2.44)			
Vessel invasion				
No	1	*0.31*		
Yes	1.27 (0.80-2.01)			
Perineural infiltration				
No	1	***0.004***	1	*0.41*
Yes	2.21 (1.29-3.78)		1.27 (0.71-2.26)	
Adyuvance				
No	1	*0.23*		
Yes	0.74 (0.45-1.20)			
POLQ staining				
HScore ≤ 10	1	***0.002***	1	***0.008***
HScore>10	2.62 (1.44-4.76)		2.27 (1.24-4.15)	

p ≤ 0.05 denotes statistical differences. p-values indicating statistical differences (p ≤ 0.05) are shown in bold.

## 4 Discussion

Recent studies have demonstrated that DNA polymerase theta, encoded by the *POLQ* gene, plays an important role in DSBs repair pathways, given its essential role in TMEJ. Previous research indicates that POLQ helps to maintain genome stability while its expression correlates with cancer progression and poor prognosis (6). Cancer cells usually show increased expression of POLQ, which would favor their survival. In contrast, normal cells express low levels or do not express POLQ (26). Many studies have also shown that POLQ activity in DNA damage repair is frequently accompanied by deletions and insertions of nucleotides (6, 11, 27, 28). Despite the mutation rate associated with POLQ, this enzyme is involved in preserving genome stability as it can be observed in its absence (12). In addition, it was shown that POLQ promotes the survival of cells deficient in homologous recombination genes, where a synthetically lethal correlation was revealed (5, 7), even very recently in pancreatic cancer cells (22, 24). The higher expression of *POLQ* mRNA observed in breast, colorectal and lung cancer, correlates with reduced patient survival (13, 15, 16, 18, 20), and may be helping cancer cells to counteract replication stress and genome instability, preventing large deletions and genomic rearrangements (29, 30). In addition, the mutations associated with TMEJ repair could serve as an adaptive mechanism favoring further clonal diversity and evolutionary resistance to treatments (31). Therefore, the differences in expression between cancer and normal tissues, and its role in survival and evolution of cancer cells have made POLQ a potential therapeutic target in cancer treatment. Furthermore, three inhibitors have been developed very recently, and although they inhibit POLQ *in vitro*, they show low potency *in vivo* and in xenograft models. One of these inhibitors targets the polymerase activity and two of them the helicase activity of POLQ (32–34).

One can say that great efforts have been made to understand how POLQ works and its functions in cells, and to develop chemical inhibitors for this unusual enzyme. On the other hand, the potential of POLQ as a biomarker in cancer remains poorly explored, aside from the mRNAs level studies that were mentioned above.

There are some reports regarding mutations in the *POLQ* gene, all found in familiar pancreatic cancer and in the undifferentiated sarcomatoid carcinoma, but both represent the minority of the cases (35–37). Likewise, not much is known regarding the relationship of *POLQ* mRNA expression and patient outcome. In agreement with our observations, two studies showed that pancreatic ductal adenocarcinoma (PDAC) patients with low *POLQ* expression have higher survival rates (22, 24). In addition, in one of them, the results indicated that this correlation is independent of KRAS status, but that there is an increase in expression of some proteins of the TMEJ pathway in pancreatic cancer cells, both in tumor samples and in cell lines carrying the activating KRAS mutation G12D (22).

Hence, in this work we evaluated the impact of POLQ in a pancreatic cancer cohort and we tested the usefulness of immunostaining as a valid technique for this type of analysis.

The data obtained in this study analyzing tumor samples by IHC from a cohort of pancreatic adenocarcinoma patients, allowed us to show that there is a strong impact of POLQ expression in the outcome of patients with this type of tumors. POLQ nuclear staining was the only variable that remained statistically significant in the multivariate analysis. Other variables such as stage and perineural infiltration, although in univariate analysis they were statistically significant, they did not remain as that in the multivariate analysis. These results indicate that POLQ may be a useful prognostic biomarker in this type of tumor, with more potential and more robust that other variables. A fact that is supported also with the results obtained from the GEPIA (**Figure 1**), that also showed the same correlation using the mRNA levels. This correlation between survival of patients with pancreatic adenocarcinoma and *POLQ* mRNA levels was also confirmed recently (22, 24). In addition, GEPIA data showed that *POLQ* mRNA is on average twenty times more abundant in pancreatic adenocarcinoma tumor samples than in normal pancreatic tissues (data not shown), which enhances the potential of POLQ as a therapeutic target and prognostic biomarker.

These findings indicate that POLQ expression is a prognostic factor of patient survival and suggest that its evaluation should be considered to guide the application of a treatment based on TMEJ inhibition. While the new POLQ inhibitors are in an early stage of development, the extensive research in this field will undoubtedly lead to the generation of new ones with higher potential. Finally, this method of POLQ analysis by IHC could be very useful for future POLQ inhibitors clinical trials.

The development of protocols to analyze POLQ expression by IHC could be helpful in other cancers, since the increase in POLQ abundance takes place in most of the tumors analyzed (7, 13–17, 19–22) and in the different tumor data included in GEPIA. In addition, IHC performed in Hepatocellular carcinoma (HCC) samples also showed that POLQ was expressed higher than in normal tissues (19). In this study they also analyzed the correlation between samples from patients with high POLQ expression in the IHC and the tumor recurrence or patient survival, finding that high POLQ expression in tumor samples predicted poorer prognosis (19). These results suggest that POLQ expression has potential as a prognostic marker in HCC when analyzed by IHC, in agreement with our results in pancreatic cancer. In the other hand, the recently published POLQ IHQ analysis performed for muscle-invasive bladder cancer patients, showed that high POLQ expression correlated with better overall survival. Although these results seem to disagree with ours, they evaluated the response to immunotherapy treatment in patients with also high PD-L1, but did not directly correlate POLQ expression and patient survival (38).

## 5 Conclusions

Our results indicated that high POLQ H-score was associated with poor survival, therefore, POLQ could be considered an independent prognostic factor for patients with pancreatic cancer.

## Data Availability

The raw data supporting the conclusions of this article will be made available by the authors, without undue reservation.

## References

[1] Sung H Ferlay J Siegel RL Laversanne M Soerjomataram I Jemal A. Global cancer statistics 2020: GLOBOCAN estimates of incidence and mortality worldwide for 36 cancers in 185 countries. CA Cancer J Clin. (2021) 71:209–49. doi: 10.3322/caac.21660 33538338

[2] McGuigan A Kelly P Turkington RC Jones C Coleman HG McCain RS. Pancreatic cancer: A review of clinical diagnosis, epidemiology, treatment and outcomes. World J Gastroenterol. (2018) 24:4846–61. doi: 10.3748/wjg.v24.i43.4846 PMC6250924 30487695

[3] Patterson-Fortin J D'Andrea AD. Exploiting the microhomology-mediated end-joining pathway in cancer therapy. Cancer Res. (2020) 80:4593–600. doi: 10.1158/0008-5472.CAN-20-1672 PMC7641946 32651257

[4] Dalmasso B Puccini A Catalano F Borea R Iaia ML Bruno W. Beyond BRCA: the emerging significance of DNA damage response and personalized treatment in pancreatic and prostate cancer patients. Int J Mol Sci. (2022) 23:4709. doi: 10.3390/ijms23094709 35563100 PMC9099822

[5] Mateos-Gomez PA Gong F Nair N Miller KM Lazzerini-Denchi E Sfeir A. Mammalian polymerase θ promotes alternative NHEJ and suppresses recombination. Nature. (2015) 518:254–7. doi: 10.1038/nature14157 PMC4718306 25642960

[6] Ramsden DA Carvajal-Garcia J Gupta GP. Mechanism, cellular functions and cancer roles of polymerase-theta-mediated DNA end joining. Nat Rev Mol Cell Biol. (2022) 23:125–40. doi: 10.1038/s41580-021-00405-2 PMC13537726 34522048

[7] Ceccaldi R Liu JC Amunugama R Hajdu I Primack B Petalcorin MI. Homologous-recombination-deficient tumours are dependent on Polθ-mediated repair. Nature. (2015) 518:258–62. doi: 10.1038/nature14184 PMC4415602 25642963

[8] Wood RD Doublié S. DNA polymerase θ (POLQ), double-strand break repair, and cancer. DNA Repair (Amst). (2016) 44:22–32. doi: 10.1016/j.dnarep.2016.05.003 27264557 PMC5114520

[9] Seki M Marini F Wood RD. POLQ (Pol theta), a DNA polymerase and DNA-dependent ATPase in human cells. Nucleic Acids Res. (2003) 31:6117–26. doi: 10.1093/nar/gkg814 PMC275456 14576298

[10] Mateos-Gomez PA Kent T Deng SK McDevitt S Kashkina E Hoang TM. The helicase domain of Polθ counteracts RPA to promote alt-NHEJ. Nat Struct Mol Biol. (2017) 24:1116–23. doi: 10.1038/nsmb.3494 PMC6047744 29058711

[11] Black SJ Ozdemir AY Kashkina E Kent T Rusanov T Ristic D. Molecular basis of microhomology-mediated end-joining by purified full-length Polθ. Nat Commun. (2019) 10:4423. doi: 10.1038/s41467-019-12272-9 31562312 PMC6764996

[12] Hwang T Reh S Dunbayev Y Zhong Y Takata Y Shen J. Defining the mutation signatures of DNA polymerase θ in cancer genomes. NAR Cancer. (2020) 2:zcaa017. doi: 10.1093/narcan/zcaa017 32885167 PMC7454005

[13] Lemée F Bergoglio V Fernandez-Vidal A MaChado-Silva A Pillaire MJ Bieth A. DNA polymerase theta up-regulation is associated with poor survival in breast cancer, perturbs DNA replication, and promotes genetic instability. Proc Natl Acad Sci U S A. (2010) 107:13390–5. doi: 10.1073/pnas.0910759107 PMC2922118 20624954

[14] Higgins GS Harris AL Prevo R Helleday T McKenna WG Buffa FM. Overexpression of POLQ confers a poor prognosis in early breast cancer patients. Oncotarget. (2010) 1:175–84. doi: 10.18632/oncotarget.v1i3 PMC2917771 20700469

[15] Pillaire MJ Selves J Gordien K Gourraud PA Gouraud PA Gentil C. A 'DNA replication' signature of progression and negative outcome in colorectal cancer. Oncogene. (2010) 29:876–87. doi: 10.1038/onc.2009.378 19901968

[16] Allera-Moreau C Rouquette I Lepage B Oumouhou N Walschaerts M Leconte E. DNA replication stress response involving PLK1, CDC6, POLQ, RAD51 and CLASPIN upregulation prognoses the outcome of early/mid-stage non-small cell lung cancer patients. Oncogenesis. (2012) 1:e30. doi: 10.1038/oncsis.2012.29 23552402 PMC3503291

[17] Shinmura K Kato H Kawanishi Y Yoshimura K Tsuchiya K Takahara Y. POLQ overexpression is associated with an increased somatic mutation load and PLK4 overexpression in lung adenocarcinoma. Cancers (Basel). (2019) 11:722. doi: 10.3390/cancers11050722 31137743 PMC6562496

[18] Rao X Xing B Wu Z Bin Y Chen Y Xu Y. Targeting polymerase θ impairs tumorigenesis and enhances radiosensitivity in lung adenocarcinoma. Cancer Sci. (2023) 114:1943–57. doi: 10.1111/cas.v114.5 PMC10154803 36642785

[19] Pan Q Wang L Liu Y Li M Zhang Y Peng W. Knockdown of POLQ interferes the development and progression of hepatocellular carcinoma through regulating cell proliferation, apoptosis and migration. Cancer Cell Int. (2021) 21:482. doi: 10.1186/s12935-021-02178-2 34517891 PMC8436534

[20] Li J Ko JM Dai W Yu VZ Ng HY Hoffmann JS. Depletion of DNA Polymerase Theta Inhibits Tumor Growth and Promotes Genome Instability through the cGAS-STING-ISG Pathway in Esophageal Squamous Cell Carcinoma. Cancers (Basel). (2021) 13:3204. doi: 10.3390/cancers13133204 34206946 PMC8268317

[21] Lessa RC Campos AH Freitas CE Silva FR Kowalski LP Carvalho AL. Identification of upregulated genes in oral squamous cell carcinomas. Head Neck. (2013) 35:1475–81. doi: 10.1002/hed.v35.10 22987617

[22] Smolinska A Singer K Golchert J Smyczynska U Fendler W Sendler M. DNA polymerase theta plays a critical role in pancreatic cancer development and metastasis. Cancers (Basel). (2022) 14:4077. doi: 10.3390/cancers14174077 36077614 PMC9454495

[23] Liu G Jin K Liu Z Su X Xu Z Li B. POLQ identifies a better response subset to immunotherapy in muscle-invasive bladder cancer with high PD-L1. Cancer Med. (2024) 13:e6962. doi: 10.1002/cam4.v13.4 38457207 PMC10922026

[24] Oh G Wang A Wang L Li J Werba G Weissinger D. POLQ inhibition elicits an immune response in homologous recombination-deficient pancreatic adenocarcinoma via cGAS/STING signaling. J Clin Invest. (2023) 133:e165934. doi: 10.1172/JCI165934 36976649 PMC10232002

[25] Tang Z Li C Kang B Gao G Zhang Z. GEPIA: a web server for cancer and normal gene expression profiling and interactive analyses. Nucleic Acids Res. (2017) 45:W98–W102. doi: 10.1093/nar/gkx247 28407145 PMC5570223

[26] Kawamura K Bahar R Seimiya M Chiyo M Wada A Okada S. DNA polymerase theta is preferentially expressed in lymphoid tissues and upregulated in human cancers. Int J Cancer. (2004) 109:9–16. doi: 10.1002/ijc.v109:1 14735462

[27] Schaub JM Soniat MM Finkelstein IJ. Polymerase theta-helicase promotes end joining by stripping single-stranded DNA-binding proteins and bridging DNA ends. Nucleic Acids Res. (2022) 50:3911–21. doi: 10.1093/nar/gkac119 PMC9023281 35357490

[28] Kent T Mateos-Gomez PA Sfeir A Pomerantz RT. Polymerase θ is a robust terminal transferase that oscillates between three different mechanisms during end-joining. Elife. (2016) 5:e13740. doi: 10.7554/eLife.13740 27311885 PMC4912351

[29] Goullet de Rugy T Bashkurov M Datti A Betous R Guitton-Sert L Cazaux C. Excess Polθ functions in response to replicative stress in homologous recombination-proficient cancer cells. Biol Open. (2016) 5:1485–92. doi: 10.1242/bio.018028 PMC5087683 27612511

[30] Feng W Simpson DA Carvajal-Garcia J Price BA Kumar RJ Mose LE. Genetic determinants of cellular addiction to DNA polymerase theta. Nat Commun. (2019) 10:4286. doi: 10.1038/s41467-019-12234-1 31537809 PMC6753077

[31] Brambati A Barry RM Sfeir A. DNA polymerase theta (Polθ) - an error-prone polymerase necessary for genome stability. Curr Opin Genet Dev. (2020) 60:119–26. doi: 10.1016/j.gde.2020.02.017 PMC7230004 32302896

[32] Bubenik M Mader P Mochirian P Vallée F Clark J Truchon JF. Identification of RP-6685, an orally bioavailable compound that inhibits the DNA polymerase activity of polθ. J Med Chem. (2022) 65:13198–215. doi: 10.1021/acs.jmedchem.2c00998 PMC9942948 36126059

[33] Zatreanu D Robinson HMR Alkhatib O Boursier M Finch H Geo L. Polθ inhibitors elicit BRCA-gene synthetic lethality and target PARP inhibitor resistance. Nat Commun. (2021) 12:3636. doi: 10.1038/s41467-021-23463-8 34140467 PMC8211653

[34] Zhou J Gelot C Pantelidou C Li A Yücel H Davis RE. A first-in-class Polymerase Theta Inhibitor selectively targets Homologous-Recombination-Deficient Tumors. Nat Cancer. (2021) 2:598–610. doi: 10.1038/s43018-021-00203-x 34179826 PMC8224818

[35] Earl J Galindo-Pumariño C Encinas J Barreto E Castillo ME Pachón V. A comprehensive analysis of candidate genes in familial pancreatic cancer families reveals a high frequency of potentially pathogenic germline variants. EBioMedicine. (2020) 53:102675. doi: 10.1016/j.ebiom.2020.102675 32113160 PMC7100610

[36] Slater EP Wilke LM Böhm LB Strauch K Lutz M Gercke N. Combinations of low-frequency genetic variants might predispose to familial pancreatic cancer. J Pers Med. (2021) 11:631. doi: 10.3390/jpm11070631 34357098 PMC8305658

[37] Gkountakos A Mafficini A Lou E Malleo G Salvia R Calicchia M. Genomic characterization of undifferentiated sarcomatoid carcinoma of the pancreas. Hum Pathol. (2022) 128:124–33. doi: 10.1016/j.humpath.2022.07.011 35850360

[38] Blattner M Liu D Robinson BD Huang D Poliakov A Gao D. SPOP mutation drives prostate tumorigenesis *in vivo* through coordinate regulation of PI3K/mTOR and AR signaling. Cancer Cell. (2017) 31:436–51. doi: 10.1016/j.ccell.2017.02.004 PMC5384998 28292441

